# Clinical usefulness of newly developed prognostic predictive score for atezolizumab plus bevacizumab for hepatocellular carcinoma

**DOI:** 10.1002/cnr2.2042

**Published:** 2024-04-05

**Authors:** Hideko Ohama, Atsushi Hiraoka, Toshifumi Tada, Masashi Hirooka, Kazuya Kariyama, Takeshi Hatanaka, Joji Tani, Koichi Takaguchi, Masanori Atsukawa, Ei Itobayashi, Takashi Nishimura, Kunihiko Tsuji, Kazuto Tajiri, Toru Ishikawa, Satoshi Yasuda, Hidenori Toyoda, Shinya Fukunishi, Chikara Ogawa, Satoru Kakizaki, Noritomo Shimada, Atsushi Naganuma, Kazuhito Kawata, Hisashi Kosaka, Hidekatsu Kuroda, Tomomitsu Matono, Yutaka Yata, Hironori Ochi, Fujimasa Tada, Kazuhiro Nouso, Asahiro Morishita, Norio Itokawa, Tomomi Okubo, Taeang Arai, Akemi Tsutsui, Takuya Nagano, Keisuke Yokohama, Hiroki Nishikawa, Michitaka Imai, Yohei Koizumi, Shinichiro Nakamura, Hiroko Iijima, Masaki Kaibori, Yoichi Hiasa, Takashi Kumada, Representing the Real‐life Practice Experts for HCC Study Group with Hepatocellular Carcinoma experts from 48 clinics in Japan (RELPEC/HCC 48 Group)

**Affiliations:** ^1^ Ehime Prefectural Central Hospital, Gastroenterology Center Matsuyama Ehime Japan; ^2^ Department of Internal Medicine Japanese Red Cross Himeji Hospital Himeji Hyogo Japan; ^3^ Department of Gastroenterology and Metabology Ehime University Graduate School of Medicine Toon Ehime Japan; ^4^ Department of Hepatology Okayama City Hospital Okayama Japan; ^5^ Department of Gastroenterology Gunma Saiseikai Maebashi Hospital Maebashi Gunma Japan; ^6^ Department of Gastroenterology and Hepatology Kagawa University Takamatsu Kagawa Japan; ^7^ Department of Hepatology Kagawa Prefectural Central Hospital Takamatsu Kagawa Japan; ^8^ Division of Gastroenterology and Hepatology, Department of Internal Medicine Nippon Medical School Tokyo Japan; ^9^ Department of Gastroenterology Asahi General Hospital Chiba Japan; ^10^ Department of Gastroenterology and Hepatology Hyogo Medical University Kochi Hyogo Japan; ^11^ Teine Keijinkai Hospital, Center of Gastroenterology Sapporo Hokkaido Japan; ^12^ Department of Gastroenterology Saiseikai Niigata Hospital Niigata Japan; ^13^ Department of Gastroenterology Toyama University Hospital Toyama Japan; ^14^ Department of Gastroenterology and Hepatology Ogaki Municipal Hospital Gifu Japan; ^15^ Department of Gastroenterology Japanese Red Cross Takamatsu Hospital Takamatsu Kagawa Japan; ^16^ Department of Clinical Research National Hospital Organization Takasaki General Medical Center Takasaki Gunma Japan; ^17^ Division of Gastroenterology and Hepatology Otakanomori Hospital Chiba Japan; ^18^ Department of Gastroenterology National Hospital Organization Takasaki General Medical Center Gunma Japan; ^19^ Hepatology Division, Department of Internal Medicine II Hamamatsu University School of Medicine Shizuoka Japan; ^20^ Department of Surgery Kansai Medical University Osaka Japan; ^21^ Division of Hepatology, Department of Internal Medicine, School of Medicine Iwate Medical University Iwate Japan; ^22^ Department of Gastroenterology Hyogo Prefectural Harima‐Himeji General Medical Center Himeji Japan; ^23^ Department of Gastroenterology Hanwa Memorial Hospital Osaka Japan; ^24^ Japanese Red Cross Matsuyama Hospital, Hepato‐biliary Center Matsuyama Ehime Japan; ^25^ Department of Gastroenterology Osaka Medical and Pharmaceutical University Osaka Japan; ^26^ Department of Nursing Gifu Kyoritsu University Gifu Japan

**Keywords:** atezolizumab plus bevacizumab, CRAFITY score, hepatocellular carcinoma, IMABALI score, IMABALI‐De score, modified albumin‐bilirubin grade, prognosis

## Abstract

**Aims:**

The aim of the present study was to elucidate detailed parameters for prediction of prognosis for patients with unresectable hepatocellular carcinoma (uHCC) receiving atezolizumab plus bevacizumab (Atez/Bev) treatment.

**Methods:**

A total of 719 patients (males 577, median age 74 years) treated with Atez/Bev between September 2020 and January 2023 were enrolled. Factors related to overall survival (OS) were extracted and a prognostic scoring system based on hazard ratio (HR) was created. OS and progression‐free survival (PFS) were retrospectively examined, and the prognostic ability of the newly developed system was compared to CRAFITY score using concordance index (c‐index) and Akaike information criterion (AIC) results.

**Results:**

Cox‐hazards multivariate analysis showed BCLC classification C/D (HR 1.4; 1 point), AFP ≥100 ng/mL (HR 1.4; 1 point), mALBI 2a (HR 1.7; 1 point), mALBI 2b/3 (HR 2.8; 2 points), and DCP ≥100 mAU/mL (HR 1.6; 1 point) as significant factors. The assigned points were added and used to develop the IMmunotherapy with AFP, BCLC staging, mALBI, and DCP evaluation (IMABALI‐De) scoring system. For IMABALI‐De scores of 0, 1, 2, 3, 4, and 5, OS was not applicable (NA), NA, 26.11, 18.79, 14.07, and 8.32 months, respectively (*p* < .001; AIC 2788.67, c‐index 0.699), while for CRAFITY scores of 0, 1, and 2, OS was 26.11, 20.29, and 11.32 months, respectively (*p* < .001; AIC 2864.54, c‐index 0.606). PFS periods for those IMABALI‐De scores were 21.75, 12.89, 9.18, 8.0, 5.0, and 3.75 months, respectively (*p* < .001; AIC 5203.32, c‐index 0.623) and for the CRAFITY scores were 10.32, 7.68, and 3.57 months, respectively (*p* < .001; AIC 5246.61, c‐index 0.574). As compared with CRAFITY score, IMABALI‐De score had better AIC and c‐index results for both OS and PFS.

**Conclusion:**

The present results indicated that the proposed IMABALI‐De score may be favorable for predicting prognosis of uHCC patients receiving Atez/Bev therapy.

## 1 INTRODUCTION

Hepatocellular carcinoma (HCC) has been reported to be the sixth most prevalent cancer type globally and third to fourth leading cause of cancer‐related deaths worldwide.^1^, ^2^ Recently, atezolizumab and bevacizumab (Atez/Bev), a recently developed immunotherapy method, received approval for treating unresectable HCC (uHCC) and has been shown to be related to favorable therapeutic outcomes in real‐world clinical practice.^3^ When possible, it is considered that Atez/Bev should be used to treat HCC patients classified as Barcelona Clinic Liver Cancer stage (BCLC)‐C, while it has also been proposed as a systemic treatment regimen for earlier stage HCC (BCLC‐B) patients showing transcatheter arterial chemoembolization (TACE) refractoriness status.^4^, ^5^ Accordingly, an assessment tool for detailed prediction of prognosis and treatment decision making is needed to provide important clinical information following introduction of Atez/Bev therapy. Previous reports have shown that c‐reactive protein (CRP) and alpha‐fetoprotein (AFP) in immunotherapy (CRAFITY) score is a useful scoring system for predicting the prognosis of patients receiving Atez/Bev.^6^, ^7^ On the other hand, the prognosis of HCC patients is known to be affected not only by tumor burden and malignant potential,^8^ but also hepatic reserve function.^9^ The aim of the present study was to develop a method for detailed prediction of prognosis for HCC patients treated with Atez/Bev based on clinical parameters related to malignancy potential, tumor burden, and hepatic function.

## 2 MATERIALS AND METHODS

**FIGURE 1:**
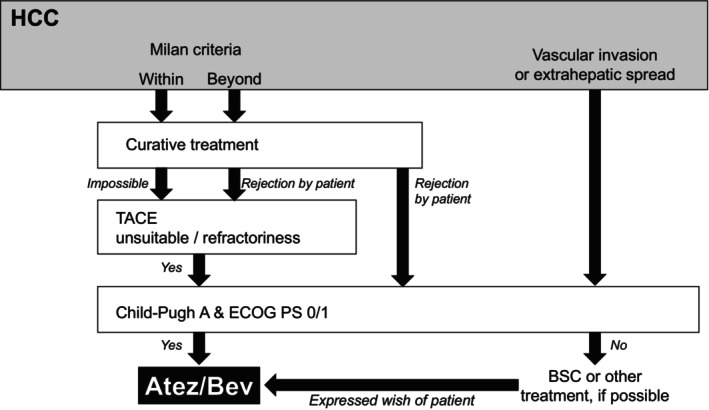
Approach for management of hepatocellular carcinoma (HCC) using atezolizumab in combination with bevacizumab (Atez/Bev). BSC, best supportive care; ECOG PS, Eastern Cooperative Oncology Group performance status; TACE, transarterial chemoembolization.

After excluding patients without data regarding CRP and/or tumor markers (AFP and/or des‐γ‐carboxy prothrombin [DCP]) (*n* = 36), 719 HCC patients treated with Atez/Bev between September 2020 and January 2023 at 25 different institutions were enrolled, after obtaining written informed consent. The treatment strategy with Atez/Bev was the same as previously reported^10^ and is illustrated in Figure 1. The choice for its administration was made by the attending physician at each medical center. Patients were given intravenous Atez/Bev, comprised of Atez (1200 mg) and Bev (15 mg/kg of body weight), in accordance with the manufacturer's recommendations, every 3 weeks.^11^ Treatment was discontinued if a serious or unacceptable adverse event (AE) occurred, or when HCC progression was noted in clinical findings. The National Cancer Institute Common Terminology Criteria for Adverse Events, version 5.0,^12^ was employed to assess AEs. The attending physician was responsible for determining the subsequent course of treatment following cessation of Atez/Bev. The present retrospective database analysis was conducted in compliance with the Japan Ministry of Health and Welfare Clinical Research Guidelines and Helsinki Declaration, following receipt of official approval.

### 2.1 Upper gastrointestinal endoscopy prior to Atez/Bev therapy

In order to mitigate the risk of gastrointestinal bleeding as an AE associated with Atez/Bev, each patient underwent an upper gastrointestinal endoscopy examination within 6 months of commencing therapy. The findings were used to monitor the presence of esophago‐gastric varices (EGV), which were categorized into three types; small straight (F1; Grade 1), enlarged tortuous (F2; Grade 2), or large coil‐shaped (F3; Grade 3). In cases where bleeding was detected and with high‐risk findings such as EGV grade 2 (F2) or higher, or positive for red‐color sign, variceal ligation, or injection sclerotherapy was performed prior to commencing Atez/Bev therapy.

### 2.2 Etiology of HCC

Using a Lumipulse HCV (FUJIREBIO Holdings, Inc., Tokyo Japan), positive anti‐HCV findings were considered to indicate that HCC was due to HCV infection, whereas HCC due to a hepatitis B virus (HBV) infection was determined when the HBV surface antigen was positive using Lumipulse HBsAg‐HQ (FUJIREBIO Holdings, Inc., Tokyo Japan). Nucleotide analog administration, such as entecavir, tenofovir, or tenofovir alafenamide fumarate, was used to treat HBV‐infected patients. For those without HBV or HCV infection but with a history of alcohol abuse (≥60 g/day), the underlying liver disease was attributed to alcohol.^13^, ^14^ Patients with a documented history of autoimmune disease were ineligible for Atez/Bev treatment.

### 2.3 Assessment of hepatic reserve function

Hepatic reserve function was assessed using Child‐Pugh classification,^15^ albumin‐bilirubin (ALBI) score,^16^, ^17^ and modified ALBI grade (mALBI), for which ALBI grade 2 is divided into two subgrades (2a and 2b) with an ALBI score of −2.27 as the cut‐off value.^9^

### 2.4 Diagnosis and evaluation of HCC stage

A diagnosis of HCC was based on the consensus statement of the Japan Society of Hepatology^18^ and tumor staging on BCLC stage.^19^ Blood test results showing increased AFP as well as contrast‐enhanced ultrasonography with perflubutane,^20^ dynamic‐CT,^21^ magnetic resonance imaging,^22^, ^23^ and/or pathological findings during the clinical course were used for diagnosis.

### 2.5 Evaluation of therapeutic efficacy of Atez/Bev treatment

To evaluate therapeutic response, the response evaluation criteria in solid tumors (RECIST), version 1.1,^24^ or modified RECIST (mRECIST)^25^ was employed. Progression‐free survival (PFS) was determined based on RECIST results. The response categories were progressive disease (PD), stable disease (SD), partial response (PR), and complete response (CR). The initial evaluation was performed approximately 6 weeks after commencing Atez/Bev treatment utilizing dynamic CT scanning. Additional dynamic CT examinations were performed when necessary, even before the 6‐week mark in some cases, depending on patient condition. Subsequent examinations were scheduled every 6 weeks following the initial assessment, with the time interval extended to 9–12 weeks after the first 6 months.^10^

### 2.6 Scoring for assessment of prognosis of uHCC patients treated with Atez/Bev

CRAFITY score^6^, ^7^ and the newly developed IMmunotherapy with AFP, BCLC staging, mALBI and DCP evaluation (IMABALI‐De) scoring system, the details of which are presented following, were used to assess the prognosis of patients with Atez/Bev. However, DCP is not available for clinical use in many regions outside of Japan, thus scoring using the IMABALI components with exclusion of DCP was also performed.

### 2.7 Statistical analysis

For statistical analyses, Cox‐hazard analysis, the Kaplan–Meier method, and log‐rank test results were utilized. Log‐rank test and Kaplan–Meier method results were used to assess overall survival (OS) and PFS following introduction of Atez/Bev. When a median value is shown, the interquartile range (IQR) is also presented. Statistical significance was determined when the *p* value was less than 0.05. Akaike information criterion (AIC) and c‐index results were used for comparisons between both prognostic predictive scores. Easy‐R, version 1.61 (Saitama Medical Center, Jichi Medical University, Saitama, Japan),^26^ a graphical user interface for R (The R Foundation for Statistical Computing, Vienna, Austria), was used to perform all statistical analyses.

## 3 RESULTS

### 3.1 Clinical features of present cohort

The fundamental characteristics of the present patients are shown in Table 1. Median age was 74 (IQR: 69–80) years and 577 were male (80.3%). The etiology of HCC was HBV in 117 (16.3%), HCV in 239 (33.2%), HBV/HCV co‐infection in one (0.1%), alcohol in 159 (22.1%), and non‐HBV‐non‐HCV‐non‐alcohol in 203 (28.2%). Among the non‐HBV non‐HCV‐non‐alcohol cases, 49 patients had metabolic dysfunction‐associated steatosis liver disease and seven primary biliary cholangitis, while the etiology in the remaining 147 could not be conclusively determined. The median ALBI score was −2.40 (mALBI grade 1:2a:2b:3 = 257:178:274:10), while median AFP was 31 (IQR 5.45–460.50) and median DCP was 280 (IQR 43.5–2996.0). Child‐Pugh scores of 5, 6, 7, 8, 9, and 10 were noted in 417 (58.0%), 224 (31.2%), 57 (7.9%), 16 (2.2%), four (0.6%), and one (0.1%), respectively, of the patients, while CRAFITY scores of 0, 1, and 2 were noted in 367 (51.0%), 274 (38.1%), and 78 (10.8%), respectively. Additionally, BCLC stage 0, A, B, C, and D was noted in 11 (1.5%), 44 (6.1%), 257 (35.7%), 390 (54.2%), and 17 (2.4%), respectively. The median duration of follow‐up examinations was 10.34 months (IQR 5.38–16.17).

**TABLE 1:** Patient characteristics.

	Total = 719
Age, years^a^	74 (69–80)
Gender, male:female	577:142
Etiology, HBV:HCV:HBV+HCV:Alc:NBNCNAlc	117:239:1:159:203
ECOG PS, 0:1:2:3:4	585:110:19:4:1
Child‐Pugh score 5:6:7:8:9:10	417:224:57:16:4:1
ALBI score^a^	−2.40 (−2.70 to −2.08)
mALBI grade 1:2a:2b:3	257:178:274:10
AST, U/L^a^	37 (27–55)
ALT, U/L^a^	27 (19–40)
Platelets, 10^4^/μL^a^	14.4 (10.7–19.55)
Total bilirubin, mg/dL^a^	0.8 (0.6–1.1)
Albumin, g/dL^a^	3.7 (3.3–4.1)
Prothrombin time, %^a^	87.0 (77.0–98.0)
AFP, ng/mL^a^	31 (5.4–460.5)
DCP, mAU/mL^a^	280 (43.5–2996.0)
BCLC stage 0:A:B:C:D	11:44:257:390:17
Treatment line, 1:2:3:4:5	487:158:47:20:7

^a^ Median (interquartile range).

Abbreviations: AFP, alpha‐fetoprotein; ALBI score, albumin‐bilirubin score; ALC, alcohol; ALT, alanine aminotransferase; AST, aspartate aminotransferase; BCLC stage, Barcelona Clinic Liver Cancer stage; DCP, des‐gamma‐carboxy prothrombin; ECOG PS, Eastern Cooperative Oncology Group performance status; HBV, hepatitis B virus; HCV, hepatitis C virus; NBNCNAlc, non‐HBV‐non‐HCV‐non‐alcohol; mALBI grade, modified ALBI grade.

### 3.2 Factor detection and construction of IMABALI‐De score

Prognostic indicators for death shown by univariate Cox‐hazard analysis findings were Eastern Cooperative Oncology Group performance status (ECOG PS) ≥1 (hazard ratio [HR] 1.597: 95% confidence interval [CI] 1.178–2.166, *p* = .003), CRP ≥1.0 mg/dL (HR 1.884: 95% CI 1.426–2.488, *p* < .001), AFP ≥100 ng/mL (HR 1.706: 95% CI 1.327–2.194, *p* < .001), and DCP ≥100 mAU/mL (HR 2.109: 95% CI 1.573–2.828, *p* < .001), as well as BCLC‐C/D (HR 1.587: 95% CI 1.219–2.067, *p* < .001), mALBI 2a (HR 1.726: 95% CI: 1.190–2.501, *p* = .004), and mALBI 2b/3 (HR 3.233: 95% CI 2.351–4.447, *p* < .001). Furthermore, prognostic indicators for death shown by multivariate analysis were AFP (≥100 ng/mL) (HR 1.392: 95% CI 1.072–1.808, *p* = .013) and DCP (≥100 mAU/mL) (HR 1.619: 95% CI 1.191–2.20, *p* = .002), as well as BCLC C/D (HR 1.352: 95% CI 1.029–1.776, *p* = .030), mALBI 2a (HR 1.749: 95% CI: 1.206–2.536, *p* = .003), and mALBI 2b/3 (HR 2.848: 95% CI 2.053–3.950, *p* < .001) (Table 2). Using multivariate Cox‐hazard analysis results, BCLC C/D, DCP ≥100 mAU/mL, AFP ≥100 ng/mL, and mALBI 2a were each assigned 1 point, while mALBI 2b/3 was given 2 points, with the sum of those point values used as IMABALI‐De score. Of the 719 enrolled patients, an IMABALI‐De score of 0 was noted in 51, a score of 1 in 115, a score of 2 in 177, a score of 3 in 169, a score of 4 in 132, and a score of 5 in 75. Findings obtained in previous reports were used to determine cut‐off values for CRP (≥1.0 mg/dL),^6^ AFP (≥100 ng/mL),^27^ and DCP (≥100 mAU/mL).^27^ When the same analysis was performed without DCP, the results were not significantly different (Supplemental Table 1). Characteristics of the patients after dividing by IMABALI‐De score are shown in Supplemental Table 2.

**TABLE 2:** Clinical factors for death shown by Cox hazard analysis.

OS	Univariate			Multivariate		
	HR	95% CI	*p* value	HR	95% CI	*p* value
Gender, female	0.732	0.518–1.035	.078			
Age ≥ 75 years	1.135	0.882–1.461	.325			
BMI ≥25 kg/m^2^	0.852	0.642–1.13	.265			
Etiology, NBNC	1.161	0.903–1.493	.245			
ECOG PS, ≥1	1.597	1.178–2.166	.003	1.112	0.796–1.553	.535
CRP ≥1.0 mg/dL	1.884	1.426–2.488	<.001	1.238	0.921–1.663	.158
AFP ≥100 ng/mL	1.706	1.327–2.194	<.001	1.392	1.072–1.808	.013
DCP ≥100 ng/mL	2.109	1.573–2.828	<.001	1.619	1.191–2.20	.002
BCLC‐C/D	1.587	1.219–2.067	<.001	1.352	1.029–1.776	.030
mALBI 2a	1.726	1.190–2.501	.004	1.749	1.206–2.536	.003
mALBI 2b/3	3.233	2.351–4.447	<.001	2.848	2.053–3.950	<.001
Atez/Bev, later line	1.163	0.901–1.502	.246			

Abbreviations: AFP, alpha‐fetoprotein; Atez/Bev, atezolizumab plus bevacizumab; BCLC, Barcelona Clinic Liver Cancer stage; BMI, body mass index; CI: confidence interval; CRP, C‐reactive protein; DCP, des‐gamma‐carboxy prothrombin; ECOG PS, Eastern Cooperative Oncology Group performance status; HR, hazard ratio; mALBI, modified albumin‐bilirubin; NBNC, non‐HBV‐non‐HCV; OS, overall survival.

### 3.3 Therapeutic response to Atez/Bev treatment according to IMABALI‐De score

Best therapeutic response was evaluated in 664 patients (IMABALI‐De score 0, 1, 2, 3, 4, 5 = 48, 108, 162, 160, 119, 67, respectively). Objective response rate (ORR) was 28.3% and disease control rate (DCR) was 78.6%. For patients with an IMABALI‐De score of 0, CR, PR, SD, and PD was noted in 3, 14, 26, and 5, respectively (ORR/DCR = 35.4%/89.5%), while the numbers for those with a score of 1 were 5, 27, 57, and 18, respectively (ORR/DCR = 29.9%/83.2%), with a score of 2 were 5, 46, 81, and 27, respectively (ORR/DCR = 32.1%/83.0%), with a score of 3 were 8, 34, 85, and 32, respectively (ORR/DCR = 26.4%/79.9%), with a score of 4 were 4, 33, 53, and 29, respectively (ORR/DCR = 31.1%/75.6%), and with a score of 5 were 0, 9, 32, and 26, respectively (ORR/DCR = 13.4%/61.2%). There was no significant difference regarding therapeutic response among the patient groups divided by IMABALI‐De score (*p* = .054).

Overall survival according to CRAFITY score, mALBI grade, Child‐Pugh score, and IMABALI‐De score.

**FIGURE 2:**
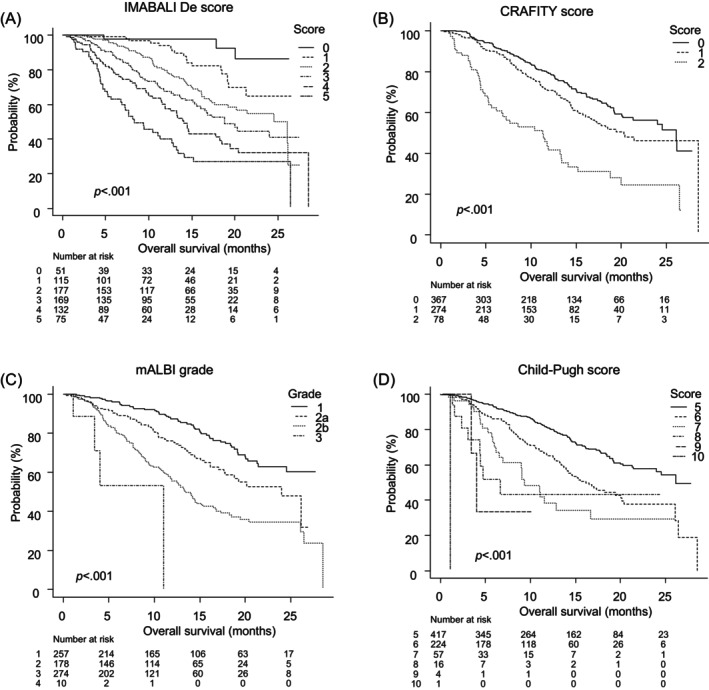
Median overall survival (OS) of patients with hepatocellular carcinoma who received Atez/Bev according to scoring method. (A) For IMABALI‐De score 0, 1, 2, 3, 4, and 5, median OS was not applicable (NA) (95% CI: NA‐NA), (95% CI:21.3‐NA), 26.11 (95% CI: 17.64‐NA), 18.79 (95% CI: 15.57‐NA), 14.07 (95% CI: 11.57–18.25), and 8.32 (95% CI: 6.79–12.86) months, respectively (*p* < .001). (B) For CRAFITY score 0, 1, and 2, median OS was 26.11 (95% CI: 20.14‐NA), 20.29 (95% CI: 16.43‐NA), and 11.32 (95% CI: 6.18–13.32) months, respectively (*p* < .001). (C) For modified albumin‐bilirubin (mALBI) grade 1, 2a, 2b, and 3, median OS was NA (95% CI: 24.54‐NA), 24.0 (95% CI: 17.75‐NA), 13.43 (95% CI: 11.82–15.32), and 11.0 (95% CI: 1.11‐NA) months, respectively (*p* < .001). (D) For Child‐Pugh score 5, 6, 7, 8, 9, and 10, median OS was 26.14 (95% CI: 24.0‐NA), 16.18 (95% CI: 13.97–20.07), 9.36 (95% CI: 6.54–16.68), 6.64 (95% CI: 3.07‐NA), 4.0 (95% CI: 3.43‐NA), and 1.11 months, respectively (*p* < .001).

Analysis based on IMABALI‐De score showed that median OS (mOS) was not applicable (NA) (95% CI: NA‐NA) for a score of 0, NA (95% CI:21.3‐NA) for a score of 1, 26.11 months (95% CI: 17.64‐NA) for a score of 2, 18.79 months (95% CI: 15.57‐NA) for a score of 3, 14.07 months (95% CI: 11.57–18.25) for a score of 4, and 8.32 months (95% CI: 6.79–12.86) for a score of 5 (*p* < .001) (Figure 2A). Interestingly, when OS was evaluated according to basal liver disease, IMABALI‐De score showed a good ability to stratify the patients into viral and nonviral groups (Supplemental Figure 1), with similar results obtained for those with either first‐ or later‐line treatment (Supplemental Figure 2). For patients with a CRAFITY score of 0, mOS was 26.11 months (95% CI: 20.14‐NA), with a score of 1 was 20.29 months (95% CI: 16.43‐NA), and with a score of 2 was 11.32 months (95% CI: 6.18–13.32) (*p* < .001) (Figure 2B). As for mALBI grade, mOS was NA (95% CI: 24.54‐NA) for grade 1, 24.0 months (95% CI: 17.75‐NA) for grade 2a, 13.43 months (95% CI: 11.82–15.32) for grade 2b, and 11.0 months (95% CI: 1.11‐NA) for grade 3 (*p* < .001) (Figure 2C). Furthermore, mOS was 26.14 months for a Child‐Pugh score of 5 (95% CI: 24.0‐NA), 16.18 months for a score of 6 (95% CI: 13.97–20.07), 9.36 months for a score of 7 (95% CI: 6.54–16.68), 6.64 months for a score of 8 (95% CI: 3.07‐NA), 4.0 months for a score of 9 (95% CI: 3.43‐NA), and 1.11 months for a score of 10 (*p* < .001) (Figure 2D). The AIC results for IMABALI‐De score, CRAFITY score, mALBI grade, and Child‐Pugh score were 2788.67, 2864.54, 2816.58, and 2829.04, respectively, while the c‐index results for those were 0.699, 0.606, 0.653, and 0.625, respectively. There were significant differences between IMABALI‐De score and CRAFITY score (*p* < .001), and between IMABALI‐De score and mALBI grade (*p* < .002) as well as Child‐Pugh score (*p* < .001). For determining OS, the present results indicate the effectiveness of IMABALI‐De score because of its better AIC results as compared with CRAFITY score, mALBI grade, and Child‐Pugh score, and also a good predictor of prognosis because of its better c‐index results.

As for IMABALI score, mOS was NA (95% CI: NA‐NA) for a score of 0, NA (95% CI: 21.32‐NA) for a score of 1, 20.39 months (95% CI: 17.32–26.14) for a score of 2, 14.61 months (95% CI: 12.5–20.43) for a score of 3, and 9.46 months (95% CI: 7.25–13.21) for a score of 4 (*p* < .001) (AIC 2799.02, c‐index 0.688) (Supplemental Figure 3).

Progression‐free survival according to CRAFITY score, mALBI grade, Child‐Pugh score, and IMABALI‐De score.

**FIGURE 3:**
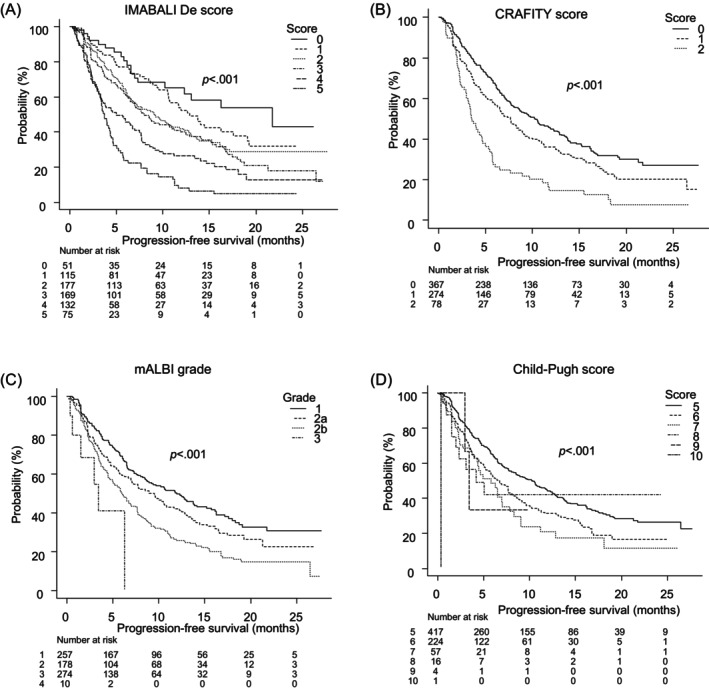
Progression‐free survival (PFS) of patients with hepatocellular carcinoma who received Atez/Bev according to scoring method. (A) For IMABALI‐De score 0, 1, 2, 3, 4, and 5, median PFS was 21.75 (95% CI: 10.21‐NA), 12.89 (95% CI: 10.61–17.57), 9.18 (95% CI: 6.68–11.79), 8.0 (95% CI: 6.54–11.0), 5.0 (95% CI: 3.46–7.0), and 3.75 (95% CI: 3.14–4.36) months, respectively (*p* < .001). (b) For CRAFITY score 0, 1, and 2, median PFS was 10.32 (95% CI: 8.11–12.25), 7.68 (95% CI: 6.25–8.96), and 3.57 (95% CI: 3.0–4.71) months, respectively (*p* < .001). (C) For mALBI grade 1, 2a, 2b, and 3, median PFS was 11.96 (95% CI: 8.64–15.21), 9.50 (95% CI: 6.96–11.64), 6.04 (95% CI: 5.0–7.0), and 3.43 (95% CI: 0.39‐NA) months, respectively (*p* < .001). (D) For Child‐Pugh score 5, 6, 7, 8, 9, and 10, median PFS was 10.32 (95% CI: 8.0–11.89), 6.68 (95% CI: 5.25–7.75), 5.79 (95% CI: 3.96–7.04), 4.18 (95% CI: 1.54‐NA), and 3.43 months, respectively (*p* < .001).

IMABALI‐De scoring showed median PFS (mPFS) of 21.75 months (95% CI: 10.21‐NA) for a score of 0, 12.89 months (95% CI: 10.61–17.57) for a score of 1, 9.18 months (95% CI: 6.68–11.79) for a score of 2, 8.0 months (95% CI: 6.54–11.0) for a score of 3, 5.0 months (95% CI: 3.46–7.0) for a score of 4, and 3.75 months (95% CI: 3.14–4.36) for a score of 5 (*p* < .001) (Figure 3A). Furthermore, when PFS was evaluated according to basal liver disease, IMABALI‐De score showed good stratification for viral and nonviral (Supplemental Figure 4), with similar results obtained for the first‐ and later‐line administration groups (Supplemental Figure 5). With CRAFITY scoring, mPFS was 10.32 months (95% CI: 8.11–12.25) for a score of 0, 7.68 months (95% CI: 6.25–8.96) for a score of 1, and 3.57 months (95% CI: 3.0–4.71) for a score of 2 (*p* < .001) (Figure 3B). As for mALBI grade, mPFS was 11.96 months (95% CI: 8.64–15.21) for grade 1, 9.50 months (95% CI: 6.96–11.64) for grade 2a, 6.04 months (95% CI: 5.0–7.0) for grade 2b, and 3.43 months (95% CI: 0.39‐NA) for grade 3 (*p* < .001) (Figure 3C). Finally, using Child‐Pugh scoring, mPFS was 10.32 months (95% CI: 8.0–11.89) for a score of 5, 6.68 months (95% CI: 5.25–7.75) for a score of 6, 5.79 months (95% CI: 3.96–7.04) for a score of 7, 4.18 months (95% CI: 1.54‐NA) for a score of 8, 3.43 months (95% CI: 3.0‐NA) for a score of 9, and 0.39 months for a score of 10 (*p* < .001) (Figure 3D). AIC values for IMABALI‐De score, CRAFITY score, mALBI grade, and Child‐Pugh score were 5203.32, 5246.24, 5245.61, and 5260.62, respectively, while c‐index values were 0.623, 0.574, 0.573, 0.559, respectively. Comparisons of ROC curves for IMABALI‐De score with those for CRAFITY score, mALBI grade or Child‐Pugh score showed significant differences (*p* < .001). In PFS, IMABALI‐De score had better AIC and c‐index results compared with CRAFITY score, mALBI grade, and Child‐Pugh score.

Using IMABALI scoring, mPFS was 16.25 months (95% CI: 11.96‐NA) for a score of 0, 9.57 months (95% CI: 7.0–12.79) for a score of 1, 8.75 months (95% CI: 7.25–12.50) for a score of 2, 6.54 months (95% CI: 4.61–7.75) for a score of 3, and 3.75 months (95% CI: 3.14–4.46) for a score of 4 (*p* < .001) (AIC 5217.7, c‐index 0.608) (Supplemental Figure 6).

## 4 DISCUSSION

Analysis of the present cohort indicated that IMABALI‐De score provided a favorable stratification of prognosis of patients with Atez/Bev. Items utilized in this scoring system for uHCC patients undergoing Atez/Bev treatment include tumor burden, that is, BCLC, and malignancy potential, that is, AFP and DCP, the same as used for CRAFITY score, while hepatic reserve function (mALBI 1, 2a, 2b, 3) is also included. That final parameter is considered to be the main reason for the superiority of IMABALI‐De as compared with CRAFITY score found in this study.

A recent report noted that both overall response rate and PFS, which had a weighted Pearson correlation coefficient of 0.71 (95% Cl = 0.52–0.84) and 0.62 (95% Cl = 0.35–0.84), respectively, demonstrated a good correlation with OS.^28^ However, post‐progression treatments have also been shown to be significant prognostic factors in regard to immunotherapy for HCC.^29^ Other studies have found that OS rates for patients who underwent post‐progression therapy after Atez/Bev failure are comparatively favorable, ranging from 74.2% to 88.2%.^30^, ^31^, ^32^ Currently, post‐progression treatment is considered to be primarily dependent on available multi‐targeting agents (MTAs), such as sorafenib, lenvatinib, regorafenib, ramucirumab, and cabozantinib, thus consideration regarding how to prolong prognosis with sequential post‐progression treatments is important. Terashima noted that post‐progression survival (PPS) had a strong relationship with OS in patients who received sorafenib treatment for HCC (*r* = .834, *p* < .001), while the PPS rate for those classified as Child‐Pugh A was better as compared with Child‐Pugh B classification (54.8 ± 17.4 vs. 32.0 ± 11.6, *p* = .015).^33^ Needless to say, better hepatic function at the time of MTA introduction is an important factor for obtaining improved prognosis following Atez/Bev failure.

For Child‐Pugh A patients, systemic therapy is mainly performed for uHCC. Over the past 30 years, a relative change in hepatic reserve function in patients with chronic liver disease has led to an increased rate, from 52.1% to 84.8%, of those classified as Child‐Pugh A,^34^ thus the necessity of an assessment tool with greater detail for clinical practice has recently been considered. Johnson et al. developed a new tool termed ALBI grade, unique because it is based on a statistical process, with findings showing clinical efficacy reported.^16^, ^17^ Furthermore, the prognostic importance of ALBI grade has been elucidated for patients HCC undergoing immunotherapy.^29^ However, ALBI grade 2 covers a wide range of patients and considered to be a clinical issue. We previously established a modified tool for assessment of hepatic function termed mALBI, in which the middle grade is divided into two sub‐grades, 2a and 2b, based on a cut‐off value of indocyanine green retention rate at 15 min (ICG‐R15) of 30%.^9^ Several reports have noted that mALBI grade has an important role to predict the prognosis of patients with HCC who undergo treatment with an MTA.^35^, ^36^, ^37^, ^38^, ^39^ In the same manner, hepatic function is also thought to be an important requirement for detailed assessment of the prognosis of HCC patients undergoing Atez/Bev therapy. Therefore, mALBI grade provides greater focus on hepatic function, along with tumor burden and HCC malignancy potential.

In addition to CRAFITY score, which focuses more on the malignant potential of HCC, mALBI grade and Child‐Pugh score, indicators of hepatic function, were also examined in the present study and the results confirmed the usefulness of IMABALI‐De score. Our research group has presented reports regarding the usefulness of nutritional indices as prognostic predictors of Atez/Bev, including NLR,^40^ prognostic nutritional index,^41^ and neo‐GPS.^42^ The present study focused on hepatic function and tumor status, thus prognostic scores predicted by nutritional indicators were not included in the comparisons, though a study will soon be conducted that includes those various scores as well as the current scoring system to determine usefulness for predicting Atez/Bev prognosis after a sufficient observation period. It should be noted that in the present multivariate analysis results, CRP, unlike neo‐GPS, did not remain a significant factor. This may be because systemic drug therapy is being introduced relatively early in an increasing number of cases, such as those classified as BCLC‐B. Therefore, it will be necessary to verify these results after incorporating a sufficient observation period in order to adequately consider this factor. In addition, specific cell subsets, such as exhausted CD8+ T and regulatory T cells, are preferentially enriched and potentially clonally expanded in HCC cases, unlike peripheral blood.^43^ Accordingly, peripheral blood fraction count alone is not necessarily a sufficient nutritional immune biomarker, indicating a need to establish a simple prognostic index combined with tumor burden, tumor malignant potential, and hepatic function.

In addition to the liver function and nutritional indices noted above, etiology, AFP reduction rate, macrovascular invasion, extrahepatic spread, AEs (skin reaction, liver damage, hypertension, proteinuria), serum interleukin‐6, granulocytes expressing PD‐1, vascular endothelial growth factor, angiopoietin‐2, insulin‐like growth factor‐1, and circulating tumor DNA have been shown to be related to the outcome of Atez/Bev treatment.^44^ Biomarkers of immune checkpoint inhibitor therapy reported include PD‐L1, ratio of tissue‐resident memory T cells to depleted CD8+ T cells in the tumor microenvironment, regulatory T cells, 11‐gene signature, high expression of CD274, T‐effector signature, and intertumoral CD8+ T cell density.^45^ The present study aimed to construct a prognostic score that is easy to use in clinical practice and does not require examination of blood or tissue‐based biomarkers. Nevertheless, the correlation of score obtained with the biomarkers presented here and other factors reported in the future will need to be examined.

Based on the clinical importance of hepatic reserve function, the present proposed scoring system is considered reasonable. Studies of BCLC staging and treatment strategy presented in 2022 recommend Atez/Bev or durvalumab plus tremelimumab (Dur/Tre) as a first‐line systemic treatment option.^46^, ^47^ The items in the IMABALI‐De scoring system, that is, tumor burden, malignant potential, and hepatic reserve function, provide a versatile system that may also be prognostically useful in Dur/Tre treatment cases. If such usefulness is shown, then this system may also be helpful for selecting systemic pharmacotherapeutic agents for uHCC.

Although therapeutic response was found to be not significantly different among the IMABALI‐De scores, except for a score of 5, PFS grading with IMABALI‐De score showed good stratification, other than between the scores of 2 and 3. We previously reported that PFS shortens as mALBI grade worsens^3^ and the present results showing a shorter duration of response as IMABALI‐De score increased may be mainly related to hepatic reserve function. During the clinical course of HCC, assessment with the present scoring system should be considered so as to not to lose the opportunity for introduction of Atez/Bev for improving prognosis within a short time after BCLC‐B status or greater is noted. While systemic therapy is the only treatment available for BCLC‐C HCC, TACE is a major option for BCLC‐B HCC as well as systemic therapy. Atez/Bev has been reported to provide clinical benefits for TACE unsuitable patients with BCLC‐B HCC beyond the up to seven criteria.^48^ Therefore, clinicians should consider switching to Atez/Bev as soon as possible, especially when the IMABALI‐De score is low, in order to obtain greater therapeutic benefit. Accordingly, the present IMABALI‐De scoring system might be useful as an indicator for switching treatment in BCLC‐B HCC patients who have initiated TACE before prediction of poor prognostic benefit of treatment with Atez/Bev.

The present study has some limitations. First, while the results were obtained from multiple institutions, they were retrospectively analyzed, thus a study with a larger number of patients will be needed in the future for validation. Additionally, because there were missing data for some of the patients, including numbers of neutrophils and lymphocyte cells, comparisons with previously reported prognostic indicators were not performed. A prospective study that uses long‐term follow‐up findings will be needed to confirm the usefulness of the present IMABALI‐De scoring system and also compare it with other prognostic indicators. It will also be important to continue to search for common clinical factors specific to combined immunotherapy treatment with Atez/Bev.

In conclusion, the proposed IMABALI‐De score, which consists of common clinical items including mALBI grade, is considered to have a good prognostic predictive ability for uHCC patients receiving treatment with Atez/Bev. With considerations based on individual case factors, introduction of Atez/Bev at the lowest possible IMABALI‐De score is recommended for improvement of prognosis.

## AUTHOR CONTRIBUTIONS

**Hideko Ohama:** Data curation (equal); formal analysis (equal); writing – original draft (equal); writing – review and editing (equal). **Atsushi Hiraoka:** Data curation (equal); investigation (equal); project administration (equal); supervision (equal); writing – review and editing (supporting). **Toshifumi Tada:** Data curation (equal). **Masashi Hirooka:** Data curation (equal). **Kazuya Kariyama:** Data curation (equal). **Takeshi Hatanaka:** Data curation (equal). **Joji Tani:** Data curation (equal). **Koichi Takaguchi:** Data curation (equal). **Masanori Atsukawa:** Data curation (equal). **Ei Itobayashi:** Data curation (equal). **Shinya Fukunishi:** Data curation (equal). **Kunihiko Tsuji:** Data curation (equal). **Kazuto Tajiri:** Data curation (equal). **Toru Ishikawa:** Data curation (equal). **Satoshi Yasuda:** Data curation (equal). **Hidenori Toyoda:** Data curation (equal). **Takashi Nishimura:** Data curation (equal). **Chikara Ogawa:** Data curation (equal). **Satoru Kakizaki:** Data curation (equal). **Noritomo Shimada:** Data curation (equal). **Atsushi Naganuma:** Data curation (equal). **Kazuhito Kawata:** Data curation (equal). **Hisashi Kosaka:** Data curation (equal). **Hidekatsu Kuroda:** Data curation (equal). **Tomomitsu Matono:** Data curation (equal). **Yutaka Yata:** Data curation (equal). **Hironori Ochi:** Supervision (supporting). **Fujimasa Tada:** Data curation (equal). **Kazuhiro Nouso:** Data curation (equal). **Asahiro Morishita:** Data curation (equal). **Norio Itokawa:** Data curation (equal). **Tomomi Okubo:** Data curation (equal). **Taeang Arai:** Data curation (equal). **Akemi Tsutsui:** Data curation (equal). **Takuya Nagano:** Data curation (equal). **Keisuke Yokohama:** Data curation (equal). **Hiroki Nishikawa:** Data curation (equal). **Michitaka Imai:** Data curation (equal). **Yohei Koizumi:** Data curation (equal). **Shinichiro Nakamura:** Data curation (equal). **Hiroko Iijima:** Data curation (equal). **Masaki Kaibori:** Data curation (equal). **Yoichi Hiasa:** Data curation (equal); supervision (supporting). **Takashi Kumada:** Data curation (equal); project administration (supporting); supervision (supporting).

## FUNDING INFORMATION

This study did not receive any external funding.

## CONFLICT OF INTEREST STATEMENT

Atsushi Hiraoka: lecture fees from Chugai, AstraZeneca, and Eli Lilly. Takashi Kumada: lecture fees from Eisai. Toshifumi Tada: lecture fees from Abbvie and Eisai. Takeshi Hatanaka: lecture fees from Eisai. None of the other authors have any potential conflicts of interest to disclose.

## ETHICS STATEMENT

Approval of research protocol. The entire study protocol was approved by the Institutional Ethics Committee of Himeji Red Cross Hospital (No. 2022‐46). After receiving official approval, this study was conducted as a retrospective analysis of database records based on the Guidelines for Clinical Research issued by the Ministry of Health and Welfare of Japan. All procedures were done in accordance with the declaration of Helsinki.

## INFORMED CONSENT

Informed consent was obtained from each patient before approval from the clinical research committee by use of an opt‐out method and written informed consent was obtained after receiving approval. Data were made anonymous before analysis to protect patient privacy.

## Supporting information


**Supplemental Table 1.** Clinical factors for death by Cox hazard analysis without data for DCP.
**Supplemental Table 2.** Patient characteristics based on IMABALI‐De score (total = 719).

**Supplemental Figure 1.** Overall survival according to IMABALI‐De score. mOS: median overall survival, 95% CI: 95% confidence interval, NA: not applicable Hepatocellular carcinoma due to (a) viral hepatitis (viral group) and (b) patients without viral hepatitis (nonviral group).
**Supplemental Figure 2.** Overall survival according to IMABALI‐De score. mOS: median overall survival, 95% CI: 95% confidence interval, NA: not applicable Hepatocellular carcinoma patients treated with (a) atezolizumab plus bevacizumab as first‐line (first line group) and (b) those that received later line (later line group) treatment.
**Supplemental Figure 3.** Overall survival according to IMABALI score. mOS: median overall survival, 95% CI: 95% confidence interval, NA: not applicable.
**Supplemental Figure 4.** Progression‐free survival according to IMABALI‐De score. mPFS: median progression‐free survival, 95% CI: 95% confidence interval, NA: not applicable Hepatocellular carcinoma due to (a) viral hepatitis (viral group) and (b) patients without viral hepatitis (nonviral group).
**Supplemental Figure 5.** Progression‐free survival according to IMABALI‐De score. mPFS: median progression‐free survival, 95% CI: 95% confidence interval, NA: not applicable (a) Hepatocellular carcinoma patients treated with atezolizumab plus bevacizumab as first‐line (first line group) and (b) those that received later line (later line group) treatment.
**Supplemental Figure 6.** Progression‐free survival according to IMABALI score. mPFS: median progression‐free survival, 95% CI: 95% confidence interval, NA: not applicable.

## Data Availability

Due to the nature of this research, the participants could not be contacted regarding whether the findings could be shared publicly, thus supporting data, including datasets generated and/or analyzed for the current study, are not publicly available.
