# Supplementary material for: Clinical usefulness of newly developed prognostic predictive score for atezolizumab plus bevacizumab for hepatocellular carcinoma
Source: Cancer Rep (Hoboken). 2024 Apr 5;7(4):e2042. doi: 10.1002/cnr2.2042 (PMC10995717; doi:10.1002/cnr2.2042)
Supplement: Supplementary file 1 — Supplemental Table 1. Clinical factors for death by Cox hazard analysis without data for DCP. Supplemental Table 2. Patient characteristics based on IMABALI‐De score (total = 719). [file CNR2-7-e2042-s001.docx]

Supplemental Table 1. Clinical factors for death by Cox hazard analysis without data for DCP

| OS | Univariate | | | Multivariate | | |
| --- | --- | --- | --- | --- | --- | --- |
|  | HR | 95% CI | *P* value | HR | 95% CI | *P* value |
| Gender, female | 0.732 | 0.518-1.035 | 0.078 |  |  |  |
| Age ≥75 years | 1.135 | 0.882-1.461 | 0.325 |  |  |  |
| BMI ≥25 kg/m^2^ | 0.852 | 0.642-1.13 | 0.265 |  |  |  |
| Etiology, NBNC | 1.161 | 0.903-1.493 | 0.245 |  |  |  |
| CRP ≥1.0 mg/dL | 1.884 | 1.426-2.488 | <0.001 | 1.315 | 0.980-1.764 | 0.07 |
| AFP ≥100 ng/mL | 1.706 | 1.327-2.194 | <0.001 | 1.531 | 1.185-1.977 | 0.001 |
| BCLC-C/D | 1.587 | 1.219-2.067 | <0.001 | 1.393 | 1.061-1.828 | 0.020 |
| mALBI 2a | 1.726 | 1.190-2.501 | 0.004 | 1.765 | 1.218-2.559 | 0.003 |
| mALBI 2b/3 | 3.233 | 2.351-4.447 | <0.001 | 2.967 | 2.141-4.111 | <0.001 |
| Atez/Bev, later line | 1.163 | 0.901-1.502 | 0.246 |  |  |  |

DCP: des-gamma-carboxy prothrombin, OS: overall survival, BMI: body mass index, NBNC: non-HBV-non-HCV, CRP: C-reactive protein, AFP: alpha-fetoprotein, BCLC: Barcelona Clinic Liver Cancer stage, mALBI: modified albumin-bilirubin, Atez/Bev: atezolizumab plus bevacizumab

Supplemental Table 2. Patient characteristics based on IMABALI-De score (total=719)

|  | IMABALI-De score | | | | | | P value |
| --- | --- | --- | --- | --- | --- | --- | --- |
|  | 0 (n=51) | 1 (n=115) | 2 (n=177) | 3 (n=169) | 4 (n=132) | 5 (n=75) |  |
| Age, years* | 75 (70-81) | 74 (68-79) | 74 (69-80) | 74 (69-80) | 74 (68-80) | 70-80) | 0.928 |
| Gender, male:female | 40:11 | 92:23 | 140:37 | 132:37 | 112:20 | 61:14 | 0.766 |
| Etiology,  HBV:HCV:HBV+HCV:  Alc:NBNCNAlc | 6:20:0:10:15 | 19:37:0:18:41 | 31:60:0:45:41 | 32:56:0:37:44 | 22:43:0:32:35 | 7:23:1:17:27 | 0.313 |
| ECOG PS, 0:1:2:3:4 | 51:0:0:0:0 | 102:11:1:0:1 | 155:18:2:2:0 | 134:31:4:0:0 | 99:27:4:2:0 | 44:23:8:0:0 | <0.001 |
| AST, U/L* | 29 (23-38) | 29 (23-40) | 33 (27-46) | 39 (29-56) | 47 (32-66) | 56 (40-87) | <0.001 |
| ALT, U/L* | 23 (23-38) | 24 (18-39) | 24 (17-38) | 27 (20-48) | 30 (21-43) | 34 (26-42) | <0.001 |
| Treatment line, first:later | 40:11 | 89:26 | 126:51 | 112:57 | 73:59 | 47:28 | 0.002 |

*Median (interquartile range). HBV: hepatitis B virus, HCV: hepatitis C virus, ALC: alcohol, NBNCNAlc: non-HBV-non-HCV-non-alcohol, ECOG PS: Eastern Cooperative Oncology Group performance status, AST: aspartate aminotransferase, ALT: alanine aminotransferase
