# Supplementary material for: Clinical usefulness of newly developed prognostic predictive score for atezolizumab plus bevacizumab for hepatocellular carcinoma
Source: Cancer Rep (Hoboken). 2024 Apr 5;7(4):e2042. doi: 10.1002/cnr2.2042 (PMC10995717; doi:10.1002/cnr2.2042)
Supplement: Supplementary file 2 — Supplemental Figure 1. Overall survival according to IMABALI‐De score. mOS: median overall survival, 95% CI: 95% confidence interval, NA: not applicable Hepatocellular carcinoma due to (a) viral hepatitis (viral group) and (b) patients without viral hepatitis (nonviral group). Supplemental Figure 2. Overall survival according to IMABALI‐De score. mOS: median overall survival, 95% CI: 95% confidence interval, NA: not applicable Hepatocellular carcinoma patients treated with (a) atezolizumab plus bevacizumab as first‐line (first line group) and (b) those that received later line (later line group) treatment. Supplemental Figure 3. Overall survival according to IMABALI score. mOS: median overall survival, 95% CI: 95% confidence interval, NA: not applicable. Supplemental Figure 4. Progression‐free survival according to IMABALI‐De score. mPFS: median progression‐free survival, 95% CI: 95% confidence interval, NA: not applicable Hepatocellular carcinoma due to (a) viral hepatitis (viral group) and (b) patients without viral hepatitis (nonviral group). Supplemental Figure 5. Progression‐free survival according to IMABALI‐De score. mPFS: median progression‐free survival, 95% CI: 95% confidence interval, NA: not applicable (a) Hepatocellular carcinoma patients treated with atezolizumab plus bevacizumab as first‐line (first line group) and (b) those that received later line (later line group) treatment. Supplemental Figure 6. Progression‐free survival according to IMABALI score. mPFS: median progression‐free survival, 95% CI: 95% confidence interval, NA: not applicable. [file CNR2-7-e2042-s002.pptx]

## Slide 1
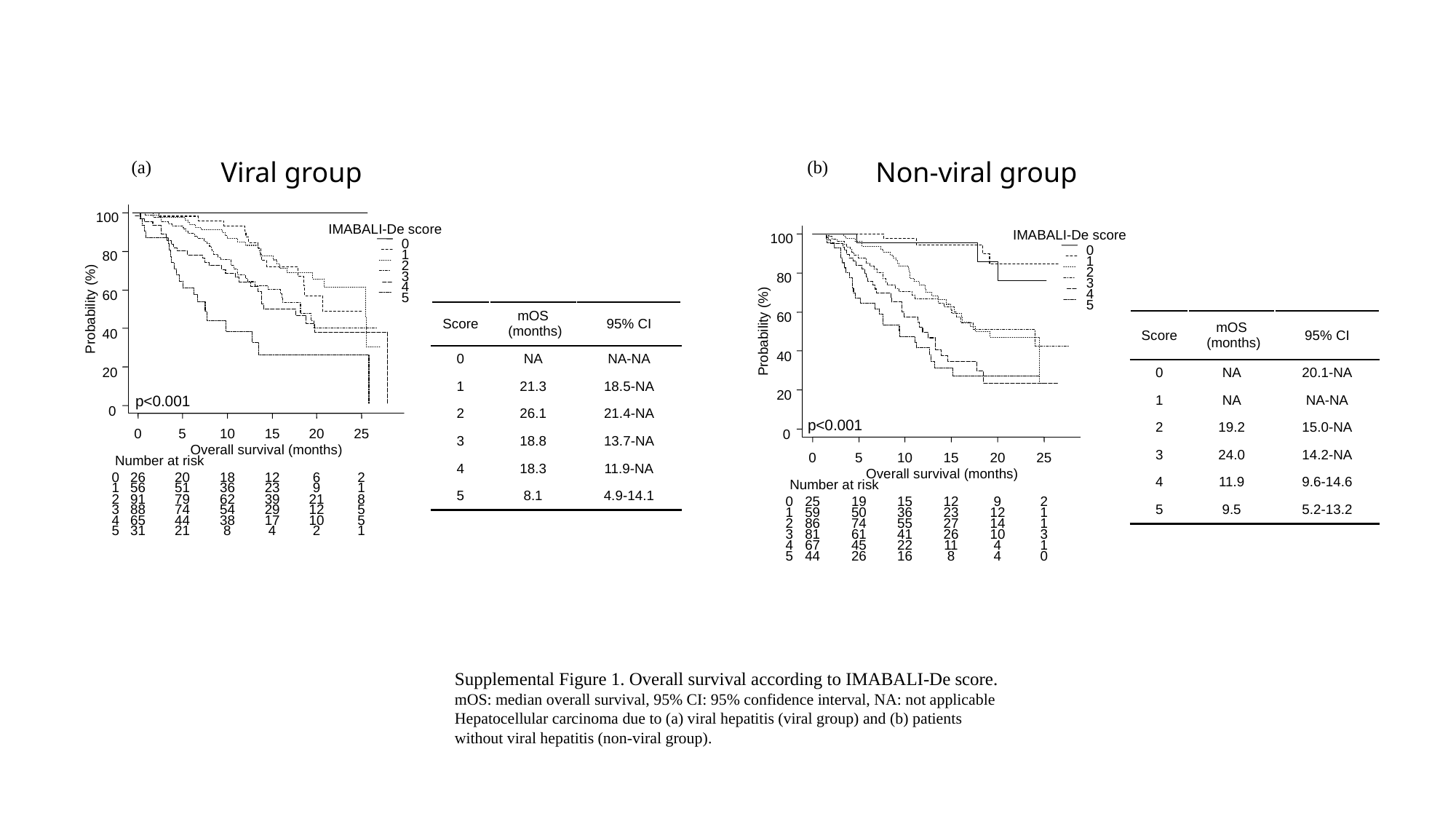

Viral group
(b)
(a)
Non-viral group
100
IMABALI-De score
0
1
2
3
4
5
80
60
Probability (%)
40
20
p<0.001
0
0
5
10
15
20
25
Overall survival (months)
Number at risk
0
26
20
18
12
6
2
1
56
51
36
23
9
1
2
91
79
62
39
21
8
3
88
74
54
29
12
5
4
65
44
38
17
10
5
5
31
21
8
4
2
1
IMABALI-De score
0
1
2
3
4
5
100
80
60
Probability (%)
40
20
0
0
5
10
15
20
25
Overall survival (months)
Number at risk
0
25
19
15
12
9
2
1
59
50
36
23
12
1
2
86
74
55
27
14
1
3
81
61
41
26
10
3
4
67
45
22
11
4
1
5
44
26
16
8
4
0
p<0.001
| Score | mOS (months) | 95% CI |
| --- | --- | --- |
| 0 | NA | NA-NA |
| 1 | 21.3 | 18.5-NA |
| 2 | 26.1 | 21.4-NA |
| 3 | 18.8 | 13.7-NA |
| 4 | 18.3 | 11.9-NA |
| 5 | 8.1 | 4.9-14.1 |
| Score | mOS (months) | 95% CI |
| --- | --- | --- |
| 0 | NA | 20.1-NA |
| 1 | NA | NA-NA |
| 2 | 19.2 | 15.0-NA |
| 3 | 24.0 | 14.2-NA |
| 4 | 11.9 | 9.6-14.6 |
| 5 | 9.5 | 5.2-13.2 |
Supplemental Figure 1. Overall survival according to IMABALI-De score.
mOS: median overall survival, 95% CI: 95% confidence interval, NA: not applicable
Hepatocellular carcinoma due to (a) viral hepatitis (viral group) and (b) patients without viral hepatitis (non-viral group).

## Slide 2
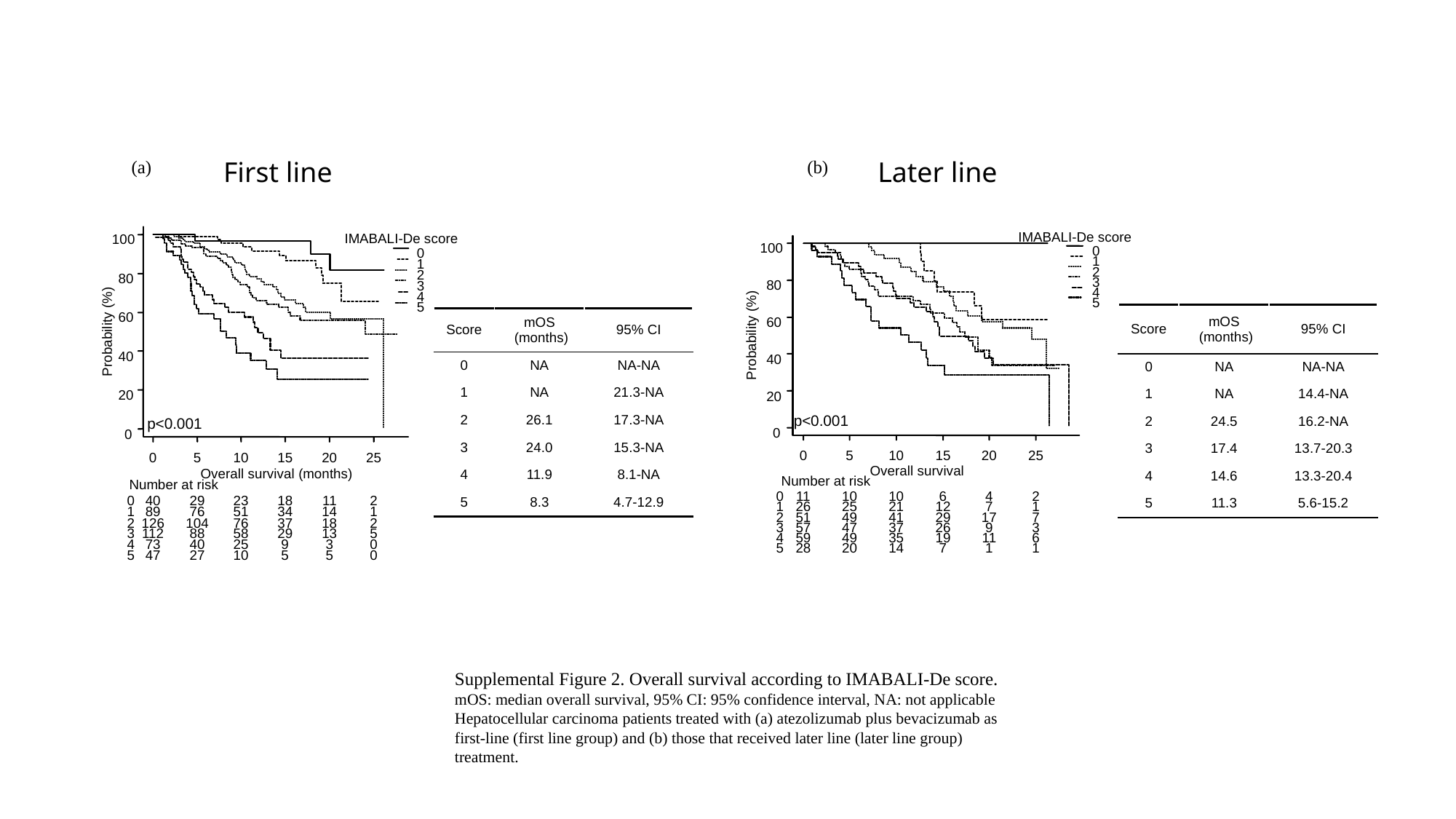

First line
(b)
(a)
Later line
IMABALI-De score
100
0
1
2
80
3
4
5
60
Probability (%)
40
20
0
0
5
10
15
20
25
Overall survival (months)
Number at risk
0
40
29
23
18
11
2
1
89
76
51
34
14
1
2
126
104
76
37
18
2
3
112
88
58
29
13
5
4
73
40
25
9
3
0
5
47
27
10
5
5
0
IMABALI-De score
100
0
1
2
3
80
4
5
60
Probability (%)
40
20
0
0
5
10
15
20
25
Overall survival
Number at risk
0
11
10
10
6
4
2
1
26
25
21
12
7
1
2
51
49
41
29
17
7
3
57
47
37
26
9
3
4
59
49
35
19
11
6
5
28
20
14
7
1
1
| Score | mOS (months) | 95% CI |
| --- | --- | --- |
| 0 | NA | NA-NA |
| 1 | NA | 14.4-NA |
| 2 | 24.5 | 16.2-NA |
| 3 | 17.4 | 13.7-20.3 |
| 4 | 14.6 | 13.3-20.4 |
| 5 | 11.3 | 5.6-15.2 |
| Score | mOS (months) | 95% CI |
| --- | --- | --- |
| 0 | NA | NA-NA |
| 1 | NA | 21.3-NA |
| 2 | 26.1 | 17.3-NA |
| 3 | 24.0 | 15.3-NA |
| 4 | 11.9 | 8.1-NA |
| 5 | 8.3 | 4.7-12.9 |
p<0.001
p<0.001
Supplemental Figure 2. Overall survival according to IMABALI-De score.
mOS: median overall survival, 95% CI: 95% confidence interval, NA: not applicable
Hepatocellular carcinoma patients treated with (a) atezolizumab plus bevacizumab as first-line (first line group) and (b) those that received later line (later line group) treatment.

## Slide 3
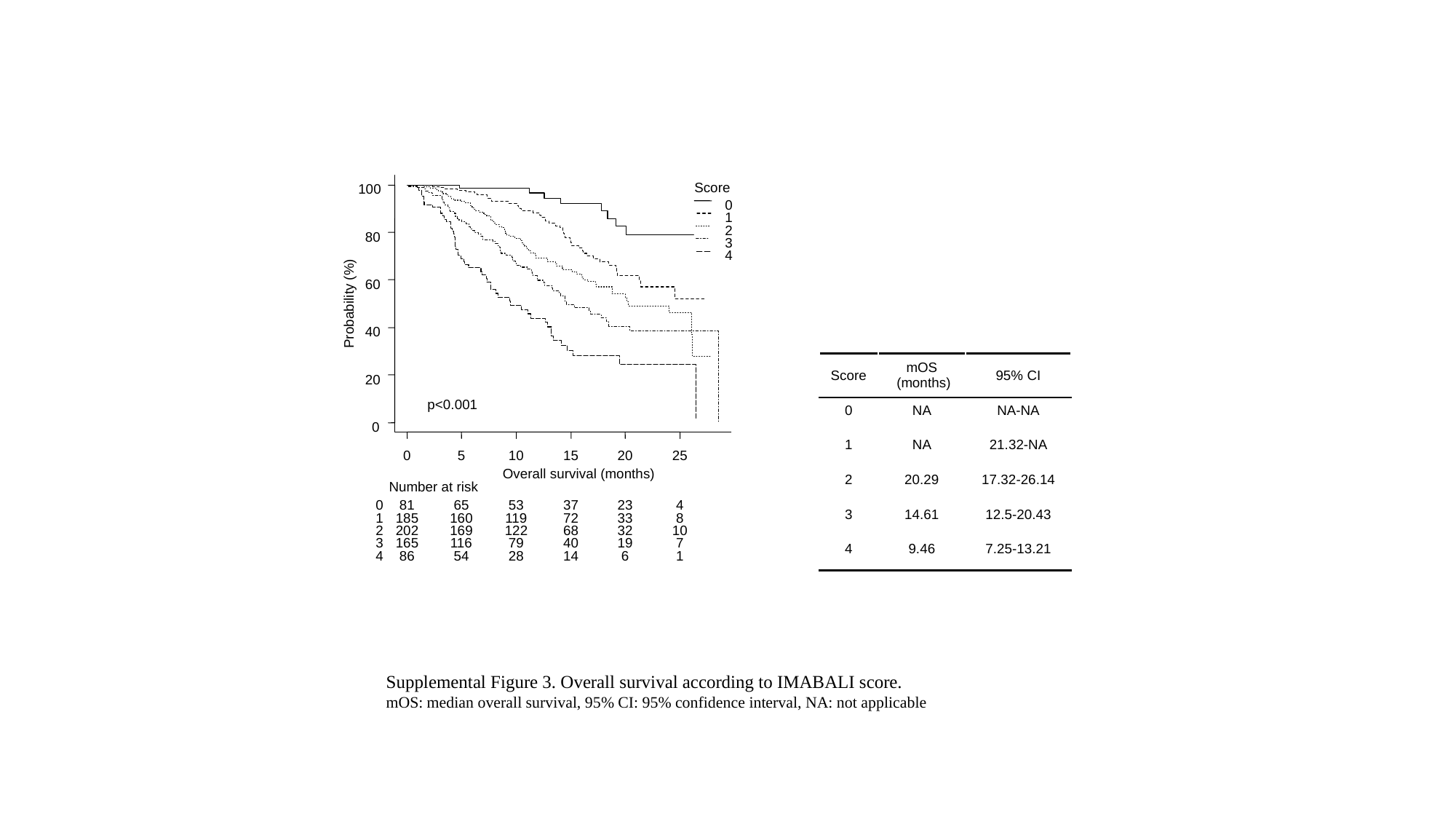

Score
0
1
2
3
4
100
80
60
Probability (%)
40
20
p<0.001
0
0
5
10
15
20
25
Overall survival (months)
Number at risk
0
81
65
53
37
23
4
1
185
160
119
72
33
8
2
202
169
122
68
32
10
3
165
116
79
40
19
7
4
86
54
28
14
6
1
| Score | mOS (months) | 95% CI |
| --- | --- | --- |
| 0 | NA | NA-NA |
| 1 | NA | 21.32-NA |
| 2 | 20.29 | 17.32-26.14 |
| 3 | 14.61 | 12.5-20.43 |
| 4 | 9.46 | 7.25-13.21 |
Supplemental Figure 3. Overall survival according to IMABALI score.
mOS: median overall survival, 95% CI: 95% confidence interval, NA: not applicable

## Slide 4
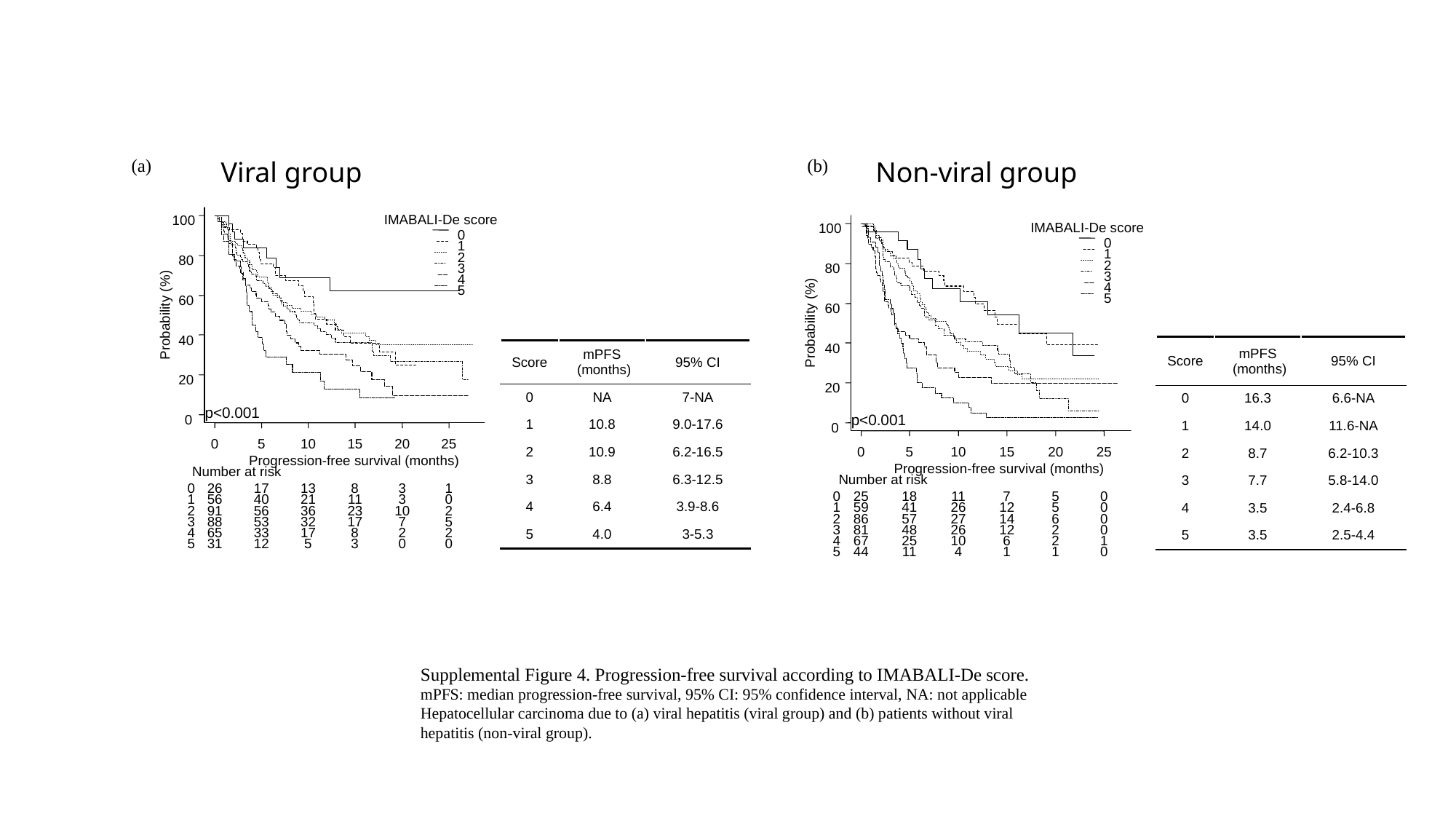

(b)
(a)
Viral group
Non-viral group
IMABALI-De score
100
0
1
2
80
3
4
5
60
Probability (%)
40
20
0
0
5
10
15
20
25
Progression-free survival (months)
Number at risk
0
26
17
13
8
3
1
1
56
40
21
11
3
0
2
91
56
36
23
10
2
3
88
53
32
17
7
5
4
65
33
17
8
2
2
5
31
12
5
3
0
0
p<0.001
IMABALI-De score
100
0
1
2
80
3
4
5
60
Probability (%)
40
20
0
0
5
10
15
20
25
Progression-free survival (months)
Number at risk
0
25
18
11
7
5
0
1
59
41
26
12
5
0
2
86
57
27
14
6
0
3
81
48
26
12
2
0
4
67
25
10
6
2
1
5
44
11
4
1
1
0
p<0.001
| Score | mPFS (months) | 95% CI |
| --- | --- | --- |
| 0 | 16.3 | 6.6-NA |
| 1 | 14.0 | 11.6-NA |
| 2 | 8.7 | 6.2-10.3 |
| 3 | 7.7 | 5.8-14.0 |
| 4 | 3.5 | 2.4-6.8 |
| 5 | 3.5 | 2.5-4.4 |
| Score | mPFS (months) | 95% CI |
| --- | --- | --- |
| 0 | NA | 7-NA |
| 1 | 10.8 | 9.0-17.6 |
| 2 | 10.9 | 6.2-16.5 |
| 3 | 8.8 | 6.3-12.5 |
| 4 | 6.4 | 3.9-8.6 |
| 5 | 4.0 | 3-5.3 |
Supplemental Figure 4. Progression-free survival according to IMABALI-De score.
mPFS: median progression-free survival, 95% CI: 95% confidence interval, NA: not applicable
Hepatocellular carcinoma due to (a) viral hepatitis (viral group) and (b) patients without viral hepatitis (non-viral group).

## Slide 5
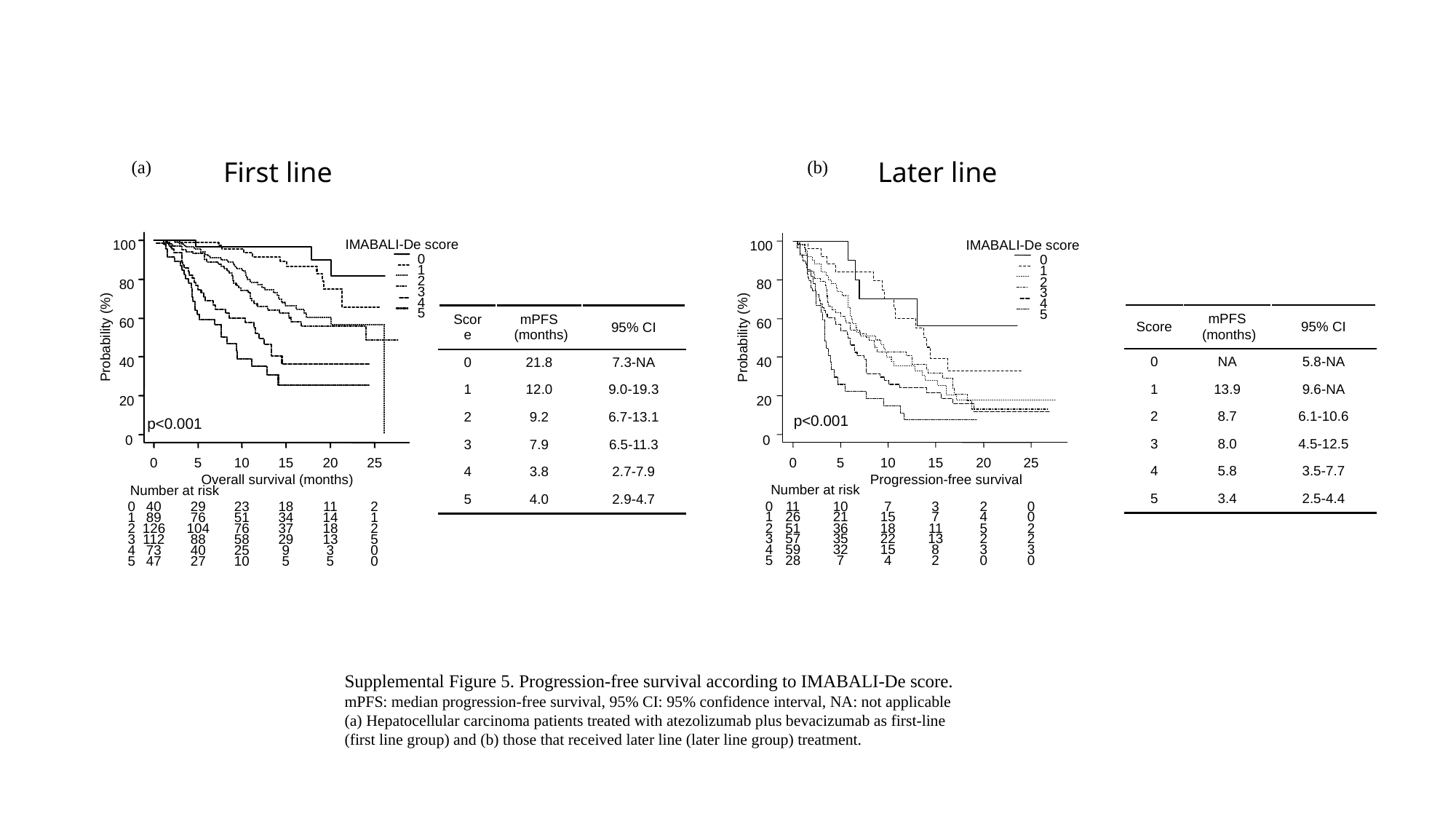

First line
(b)
(a)
Later line
IMABALI-De score
100
0
1
2
80
3
4
5
60
Probability (%)
40
20
0
0
5
10
15
20
25
Overall survival (months)
Number at risk
0
40
29
23
18
11
2
1
89
76
51
34
14
1
2
126
104
76
37
18
2
3
112
88
58
29
13
5
4
73
40
25
9
3
0
5
47
27
10
5
5
0
IMABALI-De score
100
0
1
2
80
3
4
5
60
Probability (%)
40
20
0
0
5
10
15
20
25
Progression-free survival
Number at risk
0
11
10
7
3
2
0
1
26
21
15
7
4
0
2
51
36
18
11
5
2
3
57
35
22
13
2
2
4
59
32
15
8
3
3
5
28
7
4
2
0
0
| Score | mPFS (months) | 95% CI |
| --- | --- | --- |
| 0 | NA | 5.8-NA |
| 1 | 13.9 | 9.6-NA |
| 2 | 8.7 | 6.1-10.6 |
| 3 | 8.0 | 4.5-12.5 |
| 4 | 5.8 | 3.5-7.7 |
| 5 | 3.4 | 2.5-4.4 |
| Score | mPFS (months) | 95% CI |
| --- | --- | --- |
| 0 | 21.8 | 7.3-NA |
| 1 | 12.0 | 9.0-19.3 |
| 2 | 9.2 | 6.7-13.1 |
| 3 | 7.9 | 6.5-11.3 |
| 4 | 3.8 | 2.7-7.9 |
| 5 | 4.0 | 2.9-4.7 |
p<0.001
p<0.001
Supplemental Figure 5. Progression-free survival according to IMABALI-De score.
mPFS: median progression-free survival, 95% CI: 95% confidence interval, NA: not applicable
(a) Hepatocellular carcinoma patients treated with atezolizumab plus bevacizumab as first-line (first line group) and (b) those that received later line (later line group) treatment.

## Slide 6
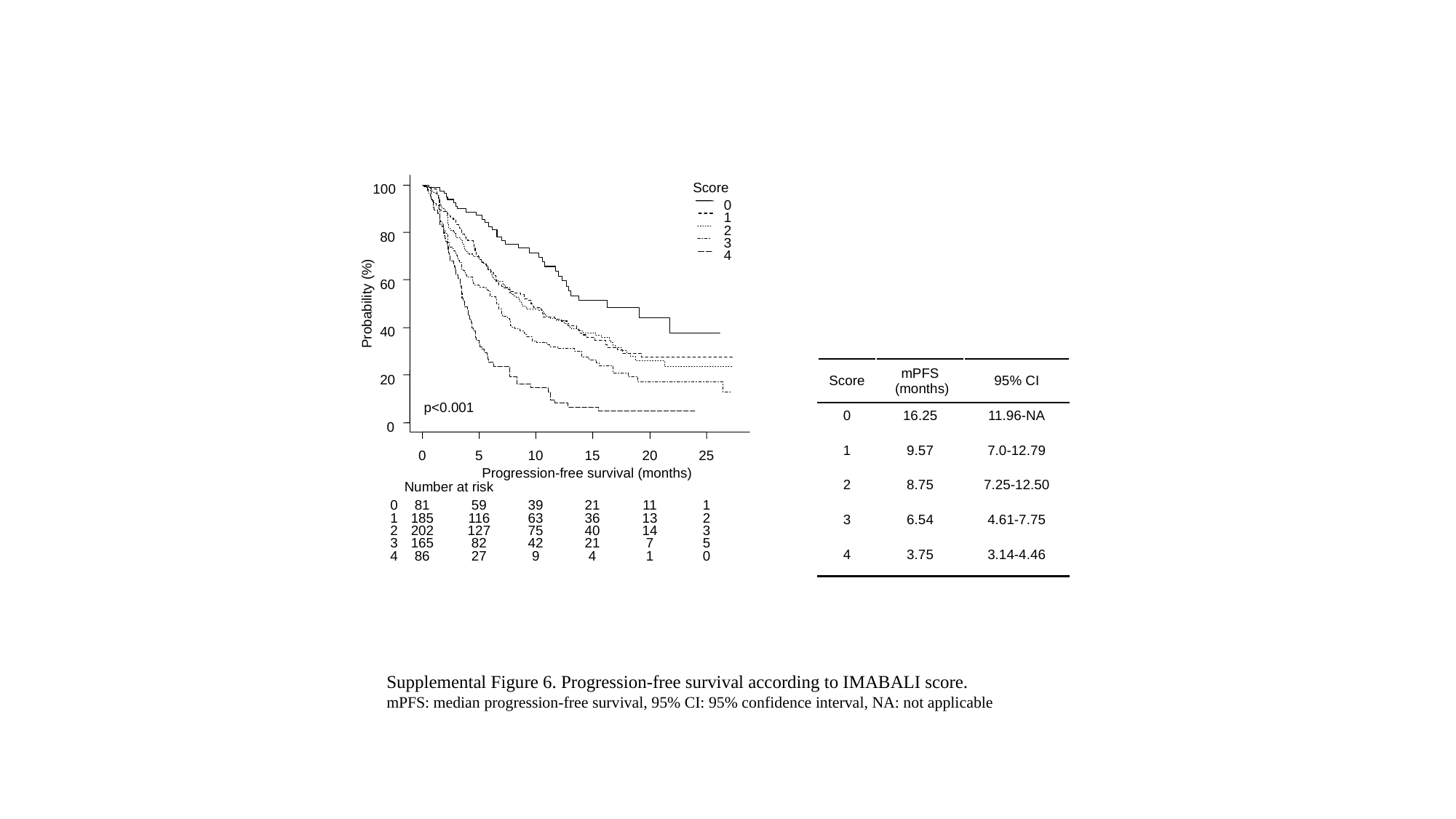

Score
100
0
1
2
80
3
4
60
Probability (%)
40
20
p<0.001
0
0
5
10
15
20
25
Progression-free survival (months)
Number at risk
0
81
59
39
21
11
1
1
185
116
63
36
13
2
2
202
127
75
40
14
3
3
165
82
42
21
7
5
4
86
27
9
4
1
0
| Score | mPFS (months) | 95% CI |
| --- | --- | --- |
| 0 | 16.25 | 11.96-NA |
| 1 | 9.57 | 7.0-12.79 |
| 2 | 8.75 | 7.25-12.50 |
| 3 | 6.54 | 4.61-7.75 |
| 4 | 3.75 | 3.14-4.46 |
Supplemental Figure 6. Progression-free survival according to IMABALI score.
mPFS: median progression-free survival, 95% CI: 95% confidence interval, NA: not applicable
